# Pilot screening for anti-HCV in adults at the *Centre Médico-Chirurgical St-Damien* in Ambanja, Madagascar

**DOI:** 10.11604/pamj.2025.52.74.49405

**Published:** 2025-10-16

**Authors:** Francesco De Maria, Jeromine Jinoro, Marie Vèronique Vavisoa, Maria Benedicth Ampilaza, Massimo Ciccozzi, Giovanni Mottini

**Affiliations:** 1 Department of Systems Medicine, “Tor Vergata” University, Rome, Italy,; 2 Centre Médico-Chirurgical St-Damien, Ambanja, Madagascar,; 3 Medical Statistics and Molecular Epidemiology Unit, University of Biomedical Campus, Rome, Italy,; 4 University Cooperation Office, University of Biomedical Campus, Rome, Italy

**Keywords:** Epidemiology, hepatitis C virus, madagascar

## To the editors of the Pan African Medical Journal

Hepatitis C virus (HCV) infection remains a significant global public health problem, particularly in resource-limited countries. The World Health Organization (WHO) has set the goal of eliminating viral hepatitis as a health threat by 2030 [1]. The most recent guidelines recommend the use of rapid tests and simplified diagnostic algorithms, adaptable to low-resource settings [2]. In sub-Saharan Africa, where a substantial proportion of the world´s HCV-infected population is estimated to live, epidemiological data remain fragmented and often outdated [3,4].

In Madagascar, knowledge on HCV remains scarce: the only published study dates back more than fifteen years and reported an anti-HCV prevalence of 1.7% in the urban population of Antananarivo [5]. We conducted a cross-sectional study at the *Centre Médico-Chirurgical St-Damien* (CMC) in Ambanja, with the aim of updating knowledge on HCV prevalence in Madagascar. Consecutive adults attending the facility were enrolled. All participants underwent a rapid test for anti-HCV. In subjects with positive results, liver ultrasound was performed on site to evaluate the presence of structural abnormalities. Virological confirmation with HCV RNA could not be performed due to economic and logistical limitations, which prevented access to advanced molecular techniques. This limitation represents a methodological weakness but reflects the operational reality typical of resource-limited countries [6]. A total of 72 adults were consecutively enrolled over the two-day survey. Two participants tested positive for anti-HCV, corresponding to a prevalence of 2.8% (95% CI: 0.8-9.6). In both cases, liver ultrasound did not reveal any structural abnormalities.

This study provides new evidence on HCV circulation in Ambanja, Madagascar, with a prevalence of 2.8%. Although the sample size is small and not representative, this figure is slightly higher than that reported in Antananarivo more than fifteen years ago (1.7%) [5]. This suggests that the infection is present and detectable outside the capital and highlights the urgency of having updated data for different regions of the country.

This study confirms this critical issue: although serology could be performed, the absence of HCV RNA testing due to economic constraints prevented diagnostic confirmation, reflecting a common scenario in resource-limited contexts [6]. As emphasized in the 2018 WHO guidelines, access to molecular testing and antiviral therapies should be a priority, even in low-income countries [7]. Liver ultrasounds of the two positive subjects were normal, suggesting the absence of advanced disease.

However, without virological confirmation, it is not possible to distinguish between past and active infection. These findings emphasize the urgency of conducting larger epidemiological studies, strengthening diagnostic capacity, and ensuring better access to care, in line with the WHO goal of eliminating viral hepatitis by 2030 [1,2,6,7]. However, conducting research in resource-limited countries such as Madagascar remains particularly challenging due to restricted access to diagnostics and treatments [8].
